# Intersections of machine learning and epidemiological methods for health services research

**DOI:** 10.1093/ije/dyaa035

**Published:** 2020-04-01

**Authors:** Sherri Rose

**Affiliations:** Department of Health Care Policy, Harvard Medical School, 180 Longwood Ave, Boston, MA, 02115, USA

**Keywords:** Machine learning, health services research, health quality, health outcomes

## Abstract

The field of health services research is broad and seeks to answer questions about the health care system. It is inherently interdisciplinary, and epidemiologists have made crucial contributions. Parametric regression techniques remain standard practice in health services research with machine learning techniques currently having low penetrance in comparison. However, studies in several prominent areas, including health care spending, outcomes and quality, have begun deploying machine learning tools for these applications. Nevertheless, major advances in epidemiological methods are also as yet underleveraged in health services research. This article summarizes the current state of machine learning in key areas of health services research, and discusses important future directions at the intersection of machine learning and epidemiological methods for health services research.

**Key Messages**

- Machine learning methods have been used less frequently in health services research, but are growing in health care spending, outcomes and quality.
- Many applied questions in health services research intersect with the methodological expertise of epidemiologists.
- Machine learning tools have promise for burgeoning areas in health services research, including methodology for difference-in-differences study designs.

## Introduction

Health services research is a broad area focused on the health care system, including costs, quality, access to providers and services, and health outcomes following care. The field benefits from the interdisciplinary expertise of health policy scholars, clinicians, health economists, statisticians and public health researchers, as well as engagement from community members, policy makers and other stakeholders. Work in health services research is also published across an array of journals. While epidemiology is a distinct discipline studying the distributions, determinants and control of health events, there is an intersection with health services research, and epidemiologists have conducted key studies in health services research.

Data sources in health services research are not typically classical epidemiological cohorts, and often use health care billing claims, registry data, surveys or electronic health records. The latter three data sources are increasingly used in epidemiology, but health care billing claims, a staple of health services research, are less common in epidemiology. Each of these data sources has well-known advantages and disadvantages,^1–3^ which will vary in importance depending on the research question.

For analysis, parametric regression techniques, rather than machine learning, are the standard in health services research. Machine learning methods aim to ‘smooth’ over the data, as traditional approaches also do, but they are often more flexible and may make fewer assumptions, typically operating in nonparametric or semiparametric models. Popular machine learning tools, such as tree-based techniques, neural networks and penalized regressions, have been used for classification questions and to identify high-risk individuals for health care interventions, but they have not been extensively integrated in health services research, especially not causal inference. The ‘promise and perils’ of these newer statistical learning tools for health services research have been discussed, with particular focus on the size of data repositories and sparsity of information.^4^^,^^5^ This article highlights several areas where machine learning has begun to advance the field of health services research, and the role of epidemiological methods at this intersection.

## Predicting health care spending

The financing of the health care system has many implications, including how health services for enrollees are provided and incentivized. Financing changes can also lead to improved health outcomes and access to care. For example, in *Better But Not Well*, authors Richard Frank and Sherry Glied discuss advances in mental health care over five decades that came not from new treatments but rather payment reforms and increased competition across providers, among other organizational changes.^6^^,^^7^ Health care spending is studied from many perspectives, including spending levels or overall growth and by health condition.^8^ The evaluation of new health payment policies is a central question in health services research and will be discussed in a later section on causality. Another impactful area is the risk adjustment of health plan payment formulas.

Plan payment risk adjustment aims to predict individual health spending Y using demographic and health condition variables X in order to reallocate funding according to the expected costs of a health plan’s enrollees. This is an attempt to disincentivize avoiding high-cost enrollees, so that market competition is geared toward efficiency and quality.^2^^,^^9^ Risk adjustment is used in many international health systems including in Belgium, Germany, The Netherlands, the USA and Israel. Epidemiologists will recognize this parametric regression problem:

$$E\left[Y|X\right]=\beta X,$$

where Y is a bounded continuous outcome. This outcome Y might be transformed before the estimation procedure using the natural log or so-called ‘top-coding’ where all high-cost enrollees above a threshold dollar amount (e.g. $250,000) are set to that threshold to improve performance with respect to specific metrics.^2^ Prediction methods for health plan payment typically focus on parametric regression, with newer economics articles developing constrained regressions where the loss function is subject to certain restrictions.

Machine learning has thus far been applied only sparingly in the plan payment risk adjustment literature, and is often published in health services journals. The regression problem for machine learning is given as:

$$E\left[Y|X\right]=f(X),$$

where f(X) is a flexible function of X, which could include discovered features in X. Three early papers in this space all considered regression trees, with one predicting payments for Medicare inpatient care,^10^ another on Medicare psychiatric payments^11^ and the last studying the addition of more complex interaction terms to predict total payments among commercially insured enrollees.^12^ Tree-based methods create sequential splits of the data based on the provided covariates (or a subset of them) to yield groupings of observations that are highly homogeneous with respect to their outcome value. These techniques have become popular due to their ability to detect interactions and other non-linear relationships among the covariates. However, tree-based methods, including aggregation methods like random forests, may overfit to the training data even when using cross-validation. I refer interested readers to an accessible introductory machine learning book for further details on tree-based methods and other statistical learning techniques.^13^

Recent plan payment risk adjustment papers implemented ensembles of various learners to predict total payments^14^^,^^15^ and mental health spending^16^ among commercially insured enrollees, in addition to new work using regression trees to discover interaction terms, this time in the Dutch risk equalization formula.^17^ Ensembles are a broad class of estimators that consider multiple algorithms to select either the single best algorithm (with respect to a particular criterion) or a weighted average of the algorithms. Tutorials on ensembles geared toward epidemiological audiences are available.^18^^,^^19^

Machine learning has also been deployed in the past 3 years in other health care spending application areas outside risk adjustment formulas. This includes demonstrating that health insurers can identify unprofitable enrollees in the unregulated United States Marketplace drug formularies, despite protections for pre-existing conditions.^20^ Other studies predicted high-cost enrollees,^21^ estimated cost-related health disparities^22^ and predicted late-life spending.^23^

Whether the machine learning approaches for health spending discussed in this section appreciably improved on standard methods varied by study, and not all compared with a traditional approach. The practical utility of machine learning versus parametric regression is context-specific and may involve assessing the prediction functions along additional metrics not included in each article (e.g. if only R^2^ was reported), as well as in external validation datasets. Evaluating algorithms using cross-validated metrics is good practice, but does not tell us how the prediction function will perform in data from subsequent years or if a prediction function created in Medicare fee-for-service enrollees is applicable to enrollees in private managed care Medicare plans.

Many ongoing practical estimation discussions surrounding health spending are centred on which variables should enter the algorithms, including the unintended consequences of incorporating social determinants of health,^2^ using more comprehensive classification systems for categorizing health conditions^12^^,^^16^ and the feasibility of integrating self-reported survey data at scale.^2^^,^^24^ Other concerns focus on how to evaluate algorithms with respect to both statistical fit and fairness to marginalized groups,^22^^,^^25^^,^^26^ and this is a major topic for future work. These considerations remain critical whether using parametric regression or machine learning. Epidemiologists’ experience with prediction methods for continuous outcomes, evaluating prediction function performance along multiple dimensions, and the social contexts of using additional demographic information would augment the interdisciplinary teams building plan payment risk adjustment formulas and health care spending algorithms.

## Predicting health outcomes and quality

Compared with health spending, there are many more examples of machine learning in health services research for the prediction of health outcomes and quality measures. A large portion, although not all, of these prediction functions consider binary outcomes, which can be written as:

$$\text{logit}(P\left[Y=1|X\right])=f(X),$$

with Y∈{0,1}. Mortality is assessed as a quality metric in some health services contexts, rather than exclusively as a health outcome. A number of recent papers have implemented machine learning to predict mortality, often among other outcomes, with respect to hospital performance.^27–30^ One paper on the increasingly popular deep neural networks looked at mortality, readmission and length of stay, but these techniques had similar classification performance to regression methods when using a similar number of covariates.^31^ Deep neural networks aim to define the strength of the associations between nodes across multiple constructed layers that form the ‘network’. Like tree-based methods, deep neutral networks may find non-linear relationships in the data and are prone to overfitting, but may additionally discover novel features. Prediction of adverse events, adherence and rates of screening, testing and visits have also been explored as quality outcomes using machine learning.^30^^,^^32–35^ Health outcomes studies have included predicting diabetes in claims data,^36^ stroke risk,^37^ obesity,^38^ postoperative pain,^39^ disease progression^40^^,^^41^ and graft failure.^42^ These health outcomes and quality studies were published across a spectrum of journals, most frequently in clinical journals.

Whereas health care quality is not a standard research question in epidemiology, health outcomes are commonly studied. Mortality prediction in particular is a frequent goal in epidemiological research, and epidemiologists’ extensive knowledge, in developing risk scores and employing calibration and discrimination measures for binary outcomes, can enhance health outcomes and quality prediction work in health services research. Notably, machine learning for time-to-event outcomes in health services work is currently scarce. Most studies discretize mortality, length of stay and other outcomes such that they are binary. For a time-to-event outcome we have T the time to outcome Y, a censoring time C, $\tilde{T}=\text{min}(T,C)$ the variable that defines which of T or C was observed earlier, and $\Delta =I(T=\tilde{T})$ an indicator for whether T was observed. The parameter of interest might be the conditional survival function $E\left[T>\theta |X\right]$ (where θ is a time point threshold) or other choice. Machine learning applications for survival are understudied in both health services research and epidemiology. Survival research questions in health services research would benefit from collaborations with epidemiologists as both fields further integrate machine learning, given the penetrance of time-to-event epidemiological methods.

I close this section by highlighting that interpretability is a frequently raised query in considering machine learning for predicting health outcomes or quality. Performance metrics such as accuracy and calibration do not capture enough information to explain how the algorithm assigned outcomes. Because applications in health services research can have significant consequences, interpretability should be a priority.^43^ Similarly, biases found in the underlying health data, including structural racism, can have massive implications if algorithms are deployed in practice.^44^ Explainability and fairness are two features found in proposed social impact statements for algorithms.^45^

## Causality, effect estimation and policy evaluation

Machine learning for causal inference is a newer area for most fields and has rarely been explored in health services research. Notable epidemiological methods development has occurred in this space, although infrequently applied. There are many causal contrasts that may be of interest, including the familiar average difference between the intervention and non-intervention groups:

$${\psi =E}_{X}\left[E\left[Y|A=1,X\right]-E\left[Y|A=0,X\right]\right],$$

where A∈{0,1} is the intervention, which could be a treatment, exposure or policy. As is well known to epidemiologists, the validity of key causal assumptions in these studies is critical. In order to define our parameters causally, we must make a series of untestable assumptions: no unmeasured confounding, consistency and no interference between subjects, (as defined under the Neyman-Rubin causal framework), among other important assumptions. We can then write:

$${\psi =E}_{X}\left[E\left[Y|A=1,X\right]-E\left[Y|A=0,X\right]\right]=E[Y^{1}-Y^{0}],$$

where $Y^{1}$ and $Y^{0}$ are the counterfactual outcomes had everyone been set to receive the intervention and not receive the intervention, respectively. The use of machine learning in causal inference estimators does not obviate the need for thoughtful construction of an underlying causal model or magically remove data quality problems.^46^

A recent health services study (published in an epidemiology journal) estimated cancer mortality risk differences by emergency department presentation with double robust machine learning.^47^ Double robust estimators will produce unbiased estimates for ψ if either the outcome regression, $E\left[Y|A,X\right],$ or the probability of being in the intervention group given covariates, $P\left[A=1|X\right],$ is estimated consistently. By incorporating machine learning into double robust methods, $E\left[Y|A,X\right]$ and $P\left[A=1|X\right]$ are estimated more flexibly and, especially when ensembles are used, minimal bias for ψ may be achieved in practice. A recent tutorial on these methods was published aimed at epidemiologists.^48^ Although not yet frequently applied, causal inference incorporating machine learning has increased in the epidemiology literature,^49–55^ with a number of studies using health care claims or electronic health record data. Issues particularly persistent in health services research with electronic health data that hinder causal inference, include missingness, misclassification and confounder selection. Variables may be collected irregularly, coding can vary by provider and facility and key confounders might be buried among hundreds of non-relevant variables. Variable selection techniques found in machine learning may aid in this last situation, but it is not guaranteed.

Comparative effectiveness research asks causal questions that consider the benefits and harms of health interventions and features substantial contributions from both health services scholars and epidemiologists.^56^^,^^57^ Frequently, comparative effectiveness involves more than two treatments with more than one parameter or contrast of interest. For example, consider a treatment that has three binary levels representing different aortic valves: $A=\left(A_{1}{,A}_{2}{,A}_{3}\right)$. Our parameters might be the three treatment-specific means:

$${\psi_{1}=E}_{X}\left[E\left[Y|A_{1}=1,X\right]\right]=E[Y_{1}^{1}],$$

 

$$\;{\psi_{2}=E}_{X}\left[E\left[Y|A_{2}=1,X\right]\right]=E[Y_{2}^{1}],$$

 

$$\;{\psi_{3}=E}_{X}\left[E\left[Y|A_{3}=1,X\right]\right]=E\left[Y_{3}^{1}\right],$$

where $Y_{1}^{1},Y_{2}^{1}$ and $Y_{3}^{1}$ are the counterfactual outcomes for having received each of the three valves, respectively. Machine learning has been examined in health services research for the comparative effectiveness of therapy, using tree-based methods in propensity score functions^58^ and a continuous treatment on traumatic brain injury with ensembles,^59^ as well as hip prosthesis on quality of life,^60^ feeding interventions in the intensive care unit^61^ and drug-eluting coronary artery stents,^62^ all using double robust machine learning. In this last study, it was demonstrated empirically that the combination of double robust estimation and machine learning likely led to the isolation of individual stent effects. Comparative effectiveness parameters have parallels to variable importance studies where we create a ranked list of effect or association parameters, and are often found in genetic epidemiology (e.g. Winham *et al.*^63^), although typically without causal assumptions. Contemporary studies for variable importance of health conditions on health care spending^8^ and ranking hospital quality based on excess mortality^64^ both used double robust machine learning.

Policy evaluation is a major facet of health services research. One prevalent design to estimate the impact of new policies is a difference-in-differences approach. The policy intervention may be implemented at a particular level of geography with several other ‘units’ at the same geographical level selected to form a comparison group. Data from before the intervention and after intervention are required to estimate the parameter of interest. This parameter is often the difference between the intervention group in the post-intervention and pre-intervention periods minus the difference between the comparison group in both time periods, hence the name ‘difference-in-differences’. The difference-in-differences parameter can be written causally and recognized as an average treatment effect among the treated:

$$\psi =E\left[Y_{\mathrm{POST}}^{1}-Y_{\mathrm{POST}}^{0}\right|A=1],$$

where the subscript *POST* represents the post-intervention time period. It is important to stress that causal interpretation of this parameter requires thoughtful consideration of the required causal assumptions.^65^ Machine learning research for difference-in-differences studies is extremely limited.^66^ However, recent work in the creation of so-called synthetic comparison groups (i.e. weighted averages among units) has incorporated machine learning (e.g. Amjad *et al*.^67^). Both parameter estimation and the construction of suitable comparison groups are vital areas for future machine learning work in policy evaluation.

## Looking forward

Health services research as a field is less flashy than many domains publicizing dramatic advances using ‘artificial intelligence’ methods, but this is not to say that careful, reasoned machine-learning work will not lead to progress in improving health care costs, quality, access, outcomes and additional areas not discussed in this piece. A focus on the external validation, generalizability and reproducibility of research results is crucial for health services findings to lead to actual successes in practice. Additionally, any time we are using data not collected for research purposes—common in health services research—we must pay extra attention to identifying the underlying processes that generated the data, which is aided by working with a diverse interdisciplinary research team.

The expertise of epidemiologists will be valuable in these teams as use of machine learning increases in health services research. This article described several areas where epidemiological methods can contribute, including causal inference, techniques for time-to-event outcomes and the inclusion of social determinants of health. However, working across disciplines is challenging. Epidemiologists may need to learn new machine learning concepts and jargon in order to communicate across these barriers, as well as additional programming languages (e.g. R and Python). Knowledge of the intricacies of the health care system is also paramount to avoid spurious results—from minutiae like changes in billing code standards to broad issues such as physician behaviour.

Growth areas for machine learning in health services research will likely encompass study designs and parameters frequently seen in the economics and policy literature, including difference-in-differences approaches discussed earlier, and instrumental variables^68^ studies. Experimental studies incorporating machine learning to reduce variance is another area.^69^ Unsupervised statistical learning methods, such as clustering, have been employed to group observations as stand-alone research questions for some time. Clustering has also been integrated into evaluations in order to study impact by groups (e.g. Lee *et al*.^70^). One consequence of the increase in the number of available variables in electronic health data resources is that evaluations conditional on algorithm-defined groups might become more common. This may be especially true for precision medicine applications and studies of treatment effect heterogeneity, two additional topics where epidemiologists have substantial insights. Machine learning also has promise for contributing to a learning health care system (e.g. Deeny and Steventon^71^). Last, data linkages across disparate sources, including imaging, wearable technology, streaming public data (e.g. Twitter) and unstructured data (e.g. text fields in electronic health records), are exciting but in need of continued, rigorous vetting.

Health services research often examines or seeks to inform policy, and machine-learning studies have strong potential to contribute to comprehensive evidence synthesis for such policy changes. Although far from comprehensive in its scope, this article has summarized key intersections between machine learning and epidemiological methods for health services research. There is great promise for progress in the future, made even more likely by further leveraging the expertise of epidemiologists.

## Funding

This work was supported by the National Institutes of Health through an NIH Director’s New Innovator Award (DP2-MD012722).

## Conflict of interest

None declared.
